# Shade Stability of Polymer-Infiltrated and Resin Nano Ceramics

**DOI:** 10.2174/1745017901814010791

**Published:** 2018-10-18

**Authors:** Martin Gabriel Schürmann, Constanze Olms

**Affiliations:** Department of Prosthodontics and Material Science, University of Leipzig, Liebigstr.12, 04103 Leipzig, Germany

**Keywords:** Shade stability, Polymer-infiltrated ceramic, Resin nano ceramic, Spectrophotometer, VITA Easyshade, VITA Enamic, Lava Ultimate

## Abstract

**Background:**

The esthetics plays an increasingly significant role in today's dentistry.

**Objective:**

The objective was to investigate the shade stability of a polymer-infiltrated and a resin nano ceramic in comparison to a conventional feldspar ceramic and an acrylate polymer.

**Methods:**

20 specimens of each of the materials, CAD-Temp (CT), Mark II (M), VITA Enamic (VE) and Lava Ultimate (LU), were prepared using the standard method. These were divided into groups (n=5) and placed in the following stain solutions: Distilled water, coffee, Coca-Cola and red wine for 14 days. The shade of each specimen was measured before and after using a spectrophotometer (n=5). The data were analyzed using ANOVA and Tukey's test (*p* ≤ 0.05).

**Results:**

The largest ΔE mean values were observed in CT by Coca-Cola (4.38 ± 0.41), in M by coffee (5.95 ± 0.62), in VE (6.02 ± 0.78) and LU (8.61 ± 0.30) by red wine. LU had the largest and strongest overall shade differences (total score: SSC=16.95) and CT the least (SSC=8.74). Overall shade differences of VE (SSC=12.03) and M (SSC=12.48) were statistically indistinguishable. After 14 days, only Coca-Cola caused clinically relevant shade differences in CT (ΔE > 2.7); this was also caused by coffee, Coca-Cola and red wine in M and coffee and red wine in VE and LU.

**Conclusion:**

On average, VE showed fewer shade differences than LU. After 14 days of immersion, shade differences which exceeded the clinical acceptance threshold of ΔE=2.7 were shown by CT in Coca-Cola, by M in coffee, Coca-Cola and red wine, and by VE and LU in coffee and red wine.

## 1 INTRODUCTION

Esthetics plays an increasingly significant role in today's dentistry. From a materials point of view, there are various denture and restoration materials. For years, tooth-colored ceramics and composites have been used. Recently, compound materials of composite and ceramic have also been used as dental restoration materials. The polymer-infiltrated ceramic VITA Enamic (VE; VITA Zahnfabrik, Bad Säckingen, Germany) and the resin nano ceramic Lava Ultimate (LU; 3M ESPE, Neuss, Germany), both available since 2012 on the dental market, are worth highlighting among the compound materials. These new materials combine positive characteristics like fracture and flexural strength [1, 2]. Altogether this leads to a high elasticity, which provides a decisive advantage on one-piece zirconia implants as a fixed connection and for implementation of the dynamic chewing processes [3, 4]. VE features an intertwined network of polymer and ceramic parts as its structure (polymer-infiltrated ceramic) and combines the mechanical properties of both ceramic and resin-based composites [5]. This polymer mesh inhibits the formation of cracks [6], thus making the material hard-wearing. LU is a resin nano ceramic consisting of about 79% nano-ceramic particles and 21% polymer. However, all materials can become discolored over time. Internal and external factors impact the shade stability. Internal factors include the matrix, the percentage of composition and distribution of the filling material, as well as the adhesive material used. External factors include exposure to the environment, UV radiation and heat, as well as colorings which occur in beverages and food, for example. These colorings can cause discoloration by adsorption and absorption [7]. The pH value of the environment can also have an influence on shade stability [8]. Some surface stains can be removed from restoration materials, especially composites, by polishing [9]. Since the material matrix plays a role in internal discolorations, methacrylates do not stain as strongly in comparison to Bis-GMA resins due to their lower viscosity and degree of water uptake [10]. Many composite restorations must be replaced because of their shade alterations [11]. Many studies on the shade stability of dental materials indicate that composites and also ceramics cannot retain their original shade when they are inserted in a stain solution [12, 13]. The CAD/CAM (Computer-Aided Design/ Computer-Aided Manufacturing) system was introduced to dentistry over 25 years ago. The low fracture rates and the long-term clinical success show the effectiveness of the system. In the beginning, ceramic blocks [14] were primarily used, and later, additional restoration materials such as composites and compounds were used. The present *in-vitro* study intends to investigate the shade stability of the polymer-infiltrated ceramic VE and the resin nano ceramic LU in comparison to the conventional feldspar ceramic Mark II (M; VITA Zahnfabrik, Bad Säckingen, Germany) and a provisional/ temporary material, the acrylate polymer CAD-Temp (CT; VITA Zahnfabrik, Bad Säckingen, Germany). These four materials, which can be all used with the CAD/CAM system, were stained by three common beverages (coffee, Coca-Cola and red wine) and distilled water over a period of 14 days. Also, these four materials can be used with one-piece zirconia implants because of a suitable E-modulus. To the best of our knowledge in the literature, there is as yet no similar study which investigates the shade stability of the two new composite materials VE and LU in comparison to the conventional dental restoration materials M and CT. The following null hypotheses were formulated for this *in-vitro* study. The feldspar ceramic shows the smallest shade differences of the four materials. All colored solutions did not stain the materials to a clinically relevant extent (ΔE > 2.7).

## 2 MATERIALS AND METHODS

Of each of the four materials CT, M, VE and LU, 20 specimens with machined surfaces were produced in a standardized manner (Table **1**).

**Fig. (1):**
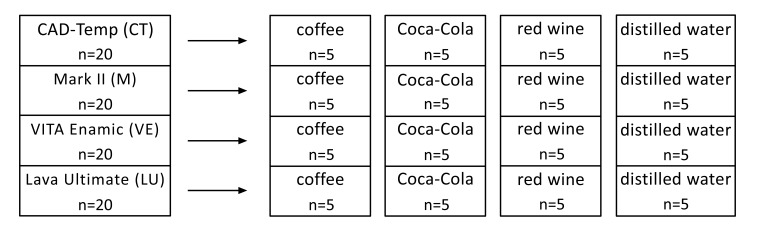
Distribution of the specimens.

Each of the materials was divided again into four equal groups (n=5). This was followed by the standardized initial shade measurement (n=5) of each specimen with the VITA Easyshade Advance 4.0 (VITA Zahnfabrik, Bad Säckingen, Germany) spectrophotometer. One group of each material (n=5) was placed in the beverages coffee (NESCAFÉ powder 2.5 g / 100ml distilled water), Coca-Cola (The Coca-Cola Company) and red wine (Blauer Zweigelt, year 2013) and a control group in distilled water over an investigation period of 14 days under standard conditions (room temperature, closed containers, darkness) (Fig. **1**).

The liquids were renewed every 3.5 days. After 14 days, the specimens were rinsed with distilled water and dried carefully with cellulose. After this, another standardized shade measurement (n=5) was conducted. The shade measurements were always conducted by the same person in a room in daylight. The measuring head (5 mm on average) of the spectrophotometer was always centrally located, lying flat on each specimen, which was placed on a white background for each shade measurement. The spectrophotometer was recalibrated after each measurement. The ΔL, Δa and Δb values between the final (day 14) and initial (day 0) shade measurements were calculated as the differences of the two-time points. The formula for the calculation of ΔE is as follows [10, 15]:

$$\Delta E=\sqrt{\Delta L^{2}+\Delta a^{2}+\Delta b^{2}}$$

Of the five ΔE values obtained for each specimen, only the mean was considered for subsequent analyses. The statistical evaluation of the data was done with IBM *SPSS* Statistics Version 21 (*SPSS* Inc., Chicago, IL, USA). The descriptive statistics (max. ΔE mean, standard deviation, sum scores) of the individual ΔL, Δa, Δb and ΔE values were collected and the data analyzed with one-way ANOVA and Tukey’s post hoc test (*p* ≤ 0.05).

## 3 RESULTS

**Fig. (2):**
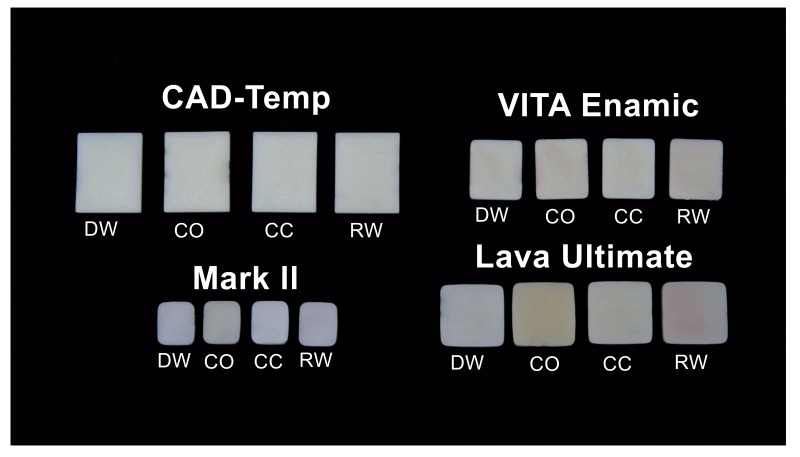
Result of the specimens after immersion. DW distilled water, CO coffee, CC Coca-Cola, RW red wine.

The tested beverages caused a change in the shade of the specimens over the period of investigation. Fig. (**2**) shows the result in the specimens (one representative example per group) after 14 days of dwell time (Fig. **2**).

The discolorations of CT by Coca-Cola and M by coffee are visible. Even more obvious are the discolorations of VE from red wine and LU from coffee and red wine. The highest ΔE mean values observed in CT were caused by Coca-Cola (4.38 ± 0.41), in M by coffee (5.95 ± 0.62), and in VE (6.02 ± 0.78) and LU (8.61 ± 0.30) by red wine. Disregarding the control group (distilled water), the smallest shade differences occurred in CT (1.75 ± 0.18) caused by red wine and in M (2.74 ± 0.65), VE (1.77 ± 0.80) and LU (1.32 ± 0.14), each caused by Coca-Cola. The total score (SSC) of the mean ΔE-values of each material of the four liquids was calculated (Table **2**).

Table **3** shows the mean ΔE-values of the four materials in each staining solution at the end of the study and non-significant differences between them (Table **3**).

LU had the largest shade differences overall (SSC=16.95) and CT the smallest (SSC=8.74). Altogether, VE (SSC=12.03) had about the same degree of shade differences as M (SSC=12.48). VE showed significantly lower ΔE values with coffee (*p* < 0.0001) and red wine (*p* = 0.001) than LU. Red wine stains LU significantly more than M (*p* < 0.0001), VE (*p* < 0.0001) and CT (*p* = 0.001). Coffee stained LU and M more significantly (*p* < 0.000) than VE and CT. CT showed significantly the greatest ΔE values with Coca-Cola in comparison to the other three materials (M: *p* < 0.002, VE: < 0.0001, LU: *p* < 0.0001). There was no detectable difference in shade between Coca-Cola and red wine for M. Coca-Cola did not stain VE and LU any more than distilled water. The acid liquid Coca-Cola stained the material CT with the greatest polymer content the most. After 14 days, only Coca-Cola caused clinically relevant shade differences (ΔE>2.7) in CT. The same was true for coffee, Coca-Cola and red wine in M, coffee and red wine in VE, and coffee and red wine in LU. Among all the materials, the acrylate polymer CT had the lowest shade differences relative to the other materials. Figs. (**3**-**6**) show the box plot charts of the ΔE values of the materials and the different stain solutions.

## 4 DISCUSSION

The objective of this *in-vitro* study was to investigate the shade stability of the polymer-infiltrated ceramic VE and the resin nano ceramic LU in comparison to the provisional acrylate polymer CT and the conventional feldspar ceramic M for significant (*p* ≤ 0.05) shade differences.

**Fig. (3):**
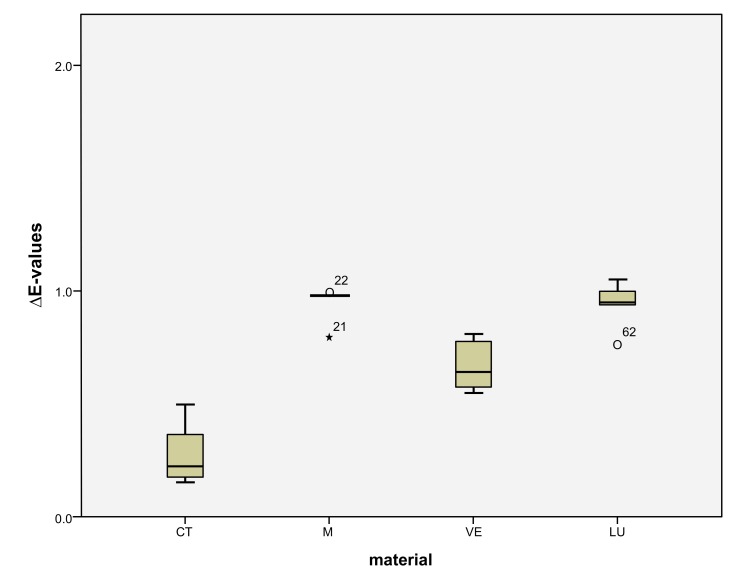
Boxplots of the der ΔE-values in distilled water. CT CAD-Temp, M Mark II, VE VITA Enamic, LU Lava Ultimate.

**Fig. (4):**
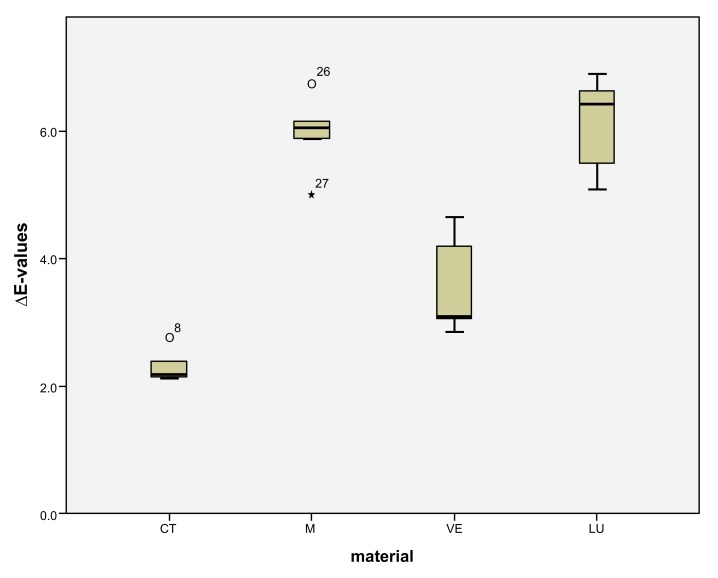
Boxplots of the der ΔE-values in coffee. CT CAD-Temp, M Mark II, VE VITA Enamic, LU Lava Ultimate.

**Fig. (5):**
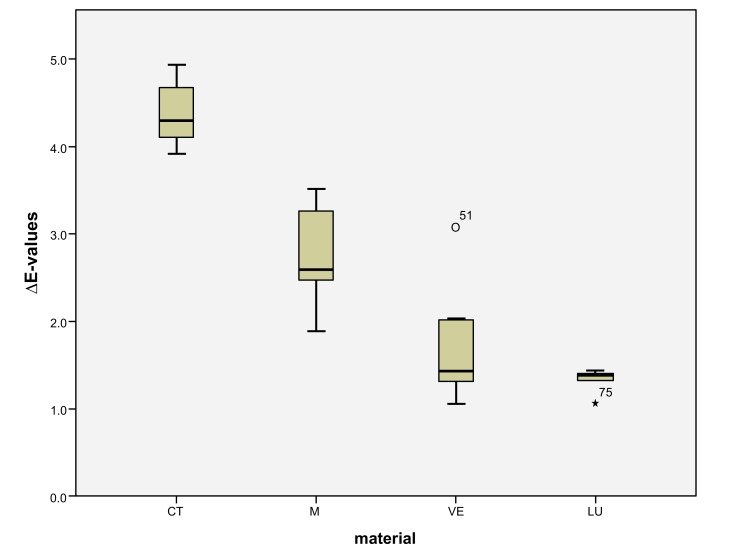
Boxplots of the der ΔE-values in Coca-Cola. CT CAD-Temp, M Mark II, VE VITA Enamic, LU Lava Ultimate.

**Fig. (6):**
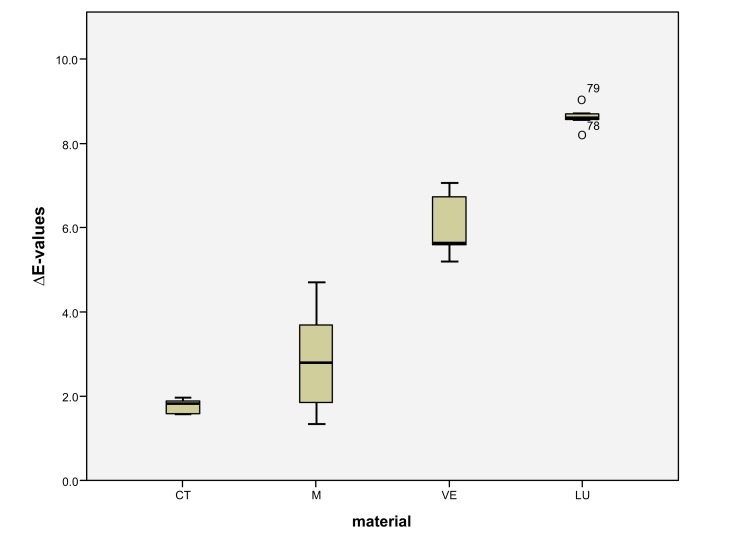
Boxplots of the der ΔE-values in red wine. CT CAD-Temp, M Mark II, VE VITA Enamic, LU Lava Ultimate.

The first null hypothesis, that the feldspar ceramic M shows the smallest shade differences between the four materials, must be rejected. M showed greater shade differences than CT in coffee (M: ΔE=5.95 ± 0.62, CT: ΔE=2.32 ± 0.26), red wine (M: ΔE=2.84 ± 1.37, CT: ΔE=1.75 ± 0.18) and distilled water (M: ΔE= 0.94 ± 0.08, CT: ΔE=0.28 ± 0.14). The total score of M (SSC=12.48) is likewise greater than that of CT (SSC=8.74). Only Coca-Cola stained CT more strongly than M (M: ΔE=2.74 ± 0.65, CT: ΔE=4.38 ± 0.41) (Figs. **3**-**6**). The second null hypothesis, that all colored solutions did not stain the materials to a clinical extent (ΔE>2.7), has to be rejected as well. CT was stained by Coca-Cola (ΔE=4.38 ± 0.41), M by coffee (ΔE=5.95 ± 0.62), by Coca-Cola (ΔE=2.74 ± 0.65) and by red wine (ΔE=2.84 ± 1.37) in a clinical extent (ΔE>2.7). There were also ΔE-values >2,7 in VE for coffee (3.56 ± 0.80) and red wine (6.02 ± 0.78) and in LU also for coffee (6.08 ± 0.76) and red wine (8.6 ± 0.30).

Now the aspects of materials and methods will be discussed.

The production of the specimens was done using standard methods from blocks from the CEREC CAD/CAM system, and each specimen of a material had the same mass. All specimens had the same thickness of 2 mm, since the thickness of the specimen could have an influence on the shade stability [16].

Coffee, Coca-Cola and red wine are everyday beverages which have frequently been used in studies investigating the shade stability of dental materials. The control group is usually immersed in distilled water or artificial saliva [10, 17-19].

The containers were sealed and darkened since the study by de Oliveira *et al*., [20] showed that a shade difference may occur in resin-based composites due to exposure to UV light and UVB radiation over a period of 5 days 20.

The investigation period of 14 days shows clearer shade differences than after only seven days. Studies show that discoloration can also increase over time after 14 days [7, 17]. The CIEL*a*b system is widely used in the literature for determining the shade of dental materials. Shade differences can be calculated with the standard formula [15].

$$\Delta E=\sqrt{\Delta L^{2}+\Delta a^{2}+\Delta b^{2}}$$

The CIEL*a*b system and the cited formula can be found in many studies on shade experiments. The spectrophotometer VITA Easyshade Advance 4.0 is excellently suited for the shade measurement of dental materials due to its ease of use, reproducibility and high precision with extra-oral specimens and intra-orally in patients [21-23]. However, some measurement errors cannot be 100% excluded by a scattering in the spectrophotometer itself or by positioning errors of the measuring head on the test specimens.

There are several studies on shade stability in these compound materials in the literature [16-18, 24-27]. The compound materials VE and LU showed good shade stability in different stain solutions over a period of 120 days in comparison to the feldspar ceramic Mark II (VITA Zahnfabrik) and other materials (VITA Hybrid-Ceramic (exp), VITA Zahnfabrik; Paradigm MZ 100, 3M ESPE; Kerr (exp), Kerr; Filtek Supreme XTE, 3M ESPE; Venus Diamond, Heraeus Kulzer; Filtek Silorane, 3M ESPE) [17]. Arocha *et al*., stated that CAD/CAM manufactured composites LU and Paragidm MZ 100 (3M ESPE) feature a greater shade stability than laboratory manufactured composites (SR Adoro, Ivoclar Vivadent AG; Premise Indirect, Kerr) [18]. In a study by Acar *et al.*, [16] the shade stability of various materials (IPS e.max CAD, Ivoclar Vivadent AG; Filtek Supreme Plus, 3M ESPE) after placement in coffee with 5,000 thermocycling cycles was investigated. In this case, the nano-hybrid composite Filtek Supreme Plus was more strongly discolored than the resin nano ceramic LU, and this in turn was more strongly discolored than the polymer-infiltrated ceramic VE. Both composite materials LU and VE showed greater shade differences than the lithium disilicate ceramic IPS e.max CAD [16]. The study by Karaokutan *et al*., studied the influence of artificial aging by a weathering machine and a one-week dwell time in distilled water on the shade stability of three ceramics using inlays. In this study, the resin nano ceramic LU showed greater shade differences than a feldspar ceramic (CEREC Blocs, Sirona Dental GmbH) or a leucite glass ceramic (IPS Empress CAD, Ivoclar Vivadent AG) [24]. In the study of Soygun *et al.,* the color stability of Lava Ultimate was not as good as the color stability of a lithium-disilicate and a leucite-ceramic after the exposition in three different mouthrinses [25]. In the study of Karakaya and Cengiz (2017), the color stability of VE specimens was better than the one of LU specimens after the immersion in coffee and red wine [26]. The study of Alharbi *et al.,* showed that VE specimens had less ΔE values than LU after the immersion in coffee, tea and red wine. Also, the residual discoloration values of VE were smaller than the ones of LU after a bleaching procedure of these stained specimens [27].

In 2014, Gómez-Polo *et al*., published, that there is no clear opinion in the literature, as to whether there is a correlation between the human eye and a spectrophotometer in tooth shade determination [28]. The use of a spectrophotometer excludes subjective mistakes of the viewer in an analogous visual shade determination with a shade template. Nevertheless, Chu *et al.,* wrote in 2010, that instrumental and visual shade measurement methods should be combined [29]. In this *in-vitro* study, the background for the shade measurements was always uniformly white, since different shades can have an effect on the shade difference ΔE [17].

There are different thresholds for the perceptibility and acceptance of tooth shade differences in the literature. Here “PT,” “50:50 PT” or “PT (50:50%)” means the perceptibility threshold at which 50% of the test subjects perceive a shade difference, and “AT,” “50:50 AT” or “AT (50:50)” means the acceptability threshold at which 50% of the test subjects accept a shade difference and would, for example, exchange or match a neighboring tooth with this shade difference.

Paravina *et al.,* defined: PT: ΔE=1.2 and AT: ΔE=2.7, where a total of 175 test subjects participated in this multi-center study [30].

The retrospective study by Khashayar *et al*., which included and compared a total of 48 studies from different databases, indicates that 44% of these studies defined PT as ΔE=1.2 and 35% of studies defined AT as ΔE=3.7 [31].

Llena *et al*., wrote that a shade difference of ΔE>3.3 is clinically unacceptable, to referring sources from the years 1991 and 2005 [10].

Ghinea *et al.,* defined PT as ΔE=1.80 and AT as ΔE=3.48 [32].

As early as 2009, Kourtis *et al*., (2009) wrote that King and deRijk (2007) had proposed the following classification for shade differences: ΔE=0 to 2: non-perceivable, ΔE=2 to 3: barely perceivable, ΔE=3 to 8: partially perceivable, ΔE>8: Perceivable [21, 33].

From the diversity on this subject in the literature, it can be concluded that the threshold values PT and AT have not been precisely defined and presumably will not be in the future. The clinically relevant threshold AT differs in the citations above by a value of 1, which is actually not perceivable as a shade difference by the human eye. Thus, a variation of the thresholds by this value is not significantly critical.

The retrospective study by Khashayar *et al*., compared several studies and most test subjects shared this determination of PT and AT [31]. Based on this study, M, VE and LU showed clinically relevant shade differences in this study in coffee, while CT did the same in Coca-Cola, and VE and LU also in red wine (ΔE>3.7). Based on the stricter values of Paravina *et al.*, M also had clinically relevant shade differences in Coca-Cola and red wine (ΔE > 2.7) [30].

With regard to the more modern composite materials, the study of Alharbi *et al*., shows that VE and LU have very good shade stability in relation to seven other tested ceramic and composite materials which are also partially suitable for the CAD/CAM system [17]. Here, VE showed even fewer shade differences than LU. In this study as well, LU had an overall lower shade stability in comparison to VE. This showed roughly the same degree of shade differences as the conventional feldspar ceramic M.

All ΔE mean values in distilled water in this study are ≤ 0.95 and therefore not clinically relevant. These are very low values for the control group in relation to the studies cited above. In the study by Erdemir *et al*., clinically relevant (ΔE > 2.7) shade differences were even detectable in composites after 6 months of dwell time in distilled water [34]. Coffee, Coca-Cola and red wine caused shade differences in the materials. Some of them were clinically relevant (ΔE > 2.7). This result was to be expected since, in many studies in the literature, comparable results occurred. The stain solutions have enough color pigments to severely stain the materials.

The individual composition of the materials is crucial for the respective shade difference. The acrylate polymer CAD-Temp showed a significantly greater shade difference in the acid stain solution Coca-Cola than the three remaining materials.

There is the consideration, that the clinically relevant shade differences primarily originate in the machined surfaces of the test specimens. This aspect should be investigated in further studies.

## CONCLUSION

Considering the limits of this *in-vitro* study, the following may be concluded. On average, the polymer-infiltrated ceramic VITA Enamic showed fewer shade differences than the resin nano ceramic Lava Ultimate.

The acrylate polymer CAD-Temp had less shade stability in acid stain solutions than the conventional feldspar ceramic Mark II, VITA Enamic and Lava Ultimate.

After 14 days of dwell time, CAD-Temp showed shade differences in Coca-Cola, Mark II in coffee, Coca-Cola and red wine, and VITA Enamic and Lava Ultimate showed shade differences in coffee and red wine, which exceeded the clinical acceptance threshold of ΔE=2.7. To investigate the aspect, that the shade differences primarily originate in the machined surfaces, there should be an accelerated artificial aging procedure of the specimens in further studies.

## Figures and Tables

**Table 1:** Used materials.

Material	Classification	Composition	Manufacturer	Dimensions [mm^3^]
CAD-Temp (CT)	acrylic polymer	acrylic polymer (86%), an organic fillers (14%)	VITA Zahnfabrik, Bad Säckingen, Germany	20 x 15 x 2
Mark II (M)	feldspathic ceramic	feldspathic ceramic	VITA Zahnfabrik, Bad Säckingen, Germany	10 x 8 x 2
VITA Enamic (VE)	polymer-infiltrated ceramic	feldspathic ceramic (86%), acrylic polymer (14%)	VITA Zahnfabrik, Bad Säckingen, Germany	14 x 12 x 2
Lava Ultimate (LU)	resin nano ceramic	resin nano ceramic (79%), polymer matrix (21%)	3M ESPE, Neuss, Germany	15 x 15 x 2

**Table 2:** Sum-scores of the mean ΔE-values.

Material	Sum-Score
CAD-Temp (CT)	8.74
Mark II (M)	12.48
VITA Enamic (VE)	12.03
Lava Ultimate (LU)	16.95

**Table 3:** Mean (standard deviation) ΔE-values. Same superscript letters in the same column and same subscript numbers in the same row indicate a non-significant difference (*p* > 0.05).

Material	Coffee Mean ΔE-Value (SD)	Coca-Cola Mean ΔE-Value (SD)	Red Wine Mean ΔE-Value (SD)	Distilled Water Mean ΔE-Value (SD)
CAD-Temp (CT)	2.32(0.26)	4.38(0.41)	1.75(0.18)^a^	0.28(0.14)
Mark II (M)	5.95(0.62)^a^	2.74(0.65)^a^_1_	2.84(1.37)^a^_1_	0.94(0.08)^a^
VITA Enamic (VE)	3.56(0.80)	1.77(0.80)^a,b^_1_	6.02(0.78)	0.66(0.11)_1_
Lava Ultimate (LU)	6.08(0.76)^a^	1.32(0.14)^b^_1_	8.61(0.30)	0.93(0.10)^a^_1_
